# Influence of SERS Activity of SnSe_2_ Nanosheets Doped with Sulfur

**DOI:** 10.3390/nano10101910

**Published:** 2020-09-24

**Authors:** Yuan Tian, Haonan Wei, Yujie Xu, Qianqian Sun, Baoyuan Man, Mei Liu

**Affiliations:** 1 School of Physics and Electronics, Shandong Normal University, Jinan 250038, China; 2018020524@stu.sdnu.edu.cn (Y.T.); 2018020530@stu.sdnu.edu.cn (H.W.); 2019020497@stu.sdnu.edu.cn (Y.X.); qianqiansun@sdnu.edu.cn (Q.S.); byman@sdnu.edu.cn (B.M.); 2 Collaborative Innovation Center of Light Manipulations and Applications, Shandong Normal University, Jinan 250358, China

**Keywords:** SnSeS, CVD, doped, SERS

## Abstract

The application of 2D semiconductor nanomaterials in the field of SERS is limited due to its weak enhancement effect and the unclear enhancement mechanism. In this study, we changed the surface morphology and energy level structure of 2D SnSe_2_ nanosheets using different amounts of S dopant. This caused the vibration coupling of the substrate and the adsorbed molecules and affects the SERS activities of the SnSe_2_ nanosheets. SERS performance of the 2D semiconductor substrate can effectively be improved by suitable doping, which can effectively break the limitation of 2D semiconductor compounds in SERS detection and will have very important significance in the fields of chemical, biological, and materials sciences. In this work, the intensities of SERS signals for R6G molecules on SnSe_0.93_S_0.94_ are 1.3 to 1.7 times stronger than those on pure SnSe_2_ substrate. It not only provides a new way to effectively improve the SERS activity of a semiconductor SERS substrates but also helps to design more efficient and stable semiconductor SERS substrates for practical application.

## 1. Introduction

Surface-enhanced Raman scattering (SERS) is an important, effective, convenient, and sensitive in situ and nondestructive method [1,2,3,4,5]. It can effectively increase the Raman signal intensity of molecules and has wide application in biomedical and biochemical detection, food security, and catalysis. It is widely accepted that there are two major mechanisms that contribute to SERS enhancement: electromagnetic mechanism (EM) and chemical mechanism (CM). However, the enhancement for semiconductor SERS, which was reported to be about 10–10^3^, is weaker than the traditional EM-enhanced SERS substrates based on some noble metal nanomaterials. It is difficult to obtain high sensitivity detection to trace molecules with a low concentration. In recent years, more and more graphene-like two-dimensional (2D) layered chalcogenide materials (LCMs), such as transition metal sulfides (MoS_2_ [6,7,8], MoSe_2_ [9,10], and WSe_2_ [11,12]), III-VIA semiconductors (GaSe [13,14], InS [15,16]), and IV-VIA semiconductors (SnS_2_ [17]) have been explored to improve the SERS performances. They have received great attention because of their outstanding optoelectronic properties, such as high charge carrier, tunable bandgap, broad absorption range, and high optical absorption. In particular, their water soluble and biocompatible properties can be widely used in SERS sensor for biomedical detection application. SnSe_2_ is a kind of IV-VI 2D LCM with a band gap energy of ~0.9 eV at room temperature [18,19]. It is widely used in the fields of solar cells [20] and atomic electrics due to its superior high external quantum efficiency (EQE) and cell performance parameters. Some studies have reported that 2D SnSe_2_ nanomaterials can be used as a good SERS substrate. The SERS limit of detection (LOD) of 2D SnSe_2_ [21] nanosheets can be compared with that of metal SERS substrate. It is a promising material to improve the SERS performances of 2D semiconductors.

However, for 2D SnSe_2_ nanomaterial, it has reached the stage in SERS where there is low enhancement of the Raman signals due to an energy level mismatch with probe molecules and incident lasers. Some studies have reported that properties of 2D layered chalcogenide composite materials can be improved or complementary to those of constituents by being tuned from one to continuously tuned with various composition. In this research, we studied the SERS activities of 2D SnSe*_x_*S*_y_* (*x* + *y* = 2) alloy nanomaterials with varying S composition using chemical vapor deposition method (CVD). SnSe_2_ and SnS_2_ are hexagonal isostructures with CdI_2_ type crystal. They are soluble in each other with minimum structural defects. For SnSe*_x_*S*_y_* alloys, the bandgap can be tuned from 1.14 to 2.08 eV depending on the S concentration [21]. The results show that 2D SnSe*_x_*S*_y_* alloys nanomaterials can be formed by using a proper ratio of Se and S sources. The coupling vibration can increase the photoinduced charge transfer (PICT) between the molecular and substrate system and improve the SERS activity of the substrate.

In this work, the intensities of SERS signals for R6G molecules on SnSe_0.93_S_0.94_ are 1.3 to 1.7 times stronger than those on pure SnSe_2_ substrate. For R6G aqueous solution, the Raman signals can be observed as low as 10^−7^ M on SnSe_0.93_S_0.94_ nanosheet substrates. The different magnitudes of enhancement for different vibrational modes were consistent with the enhancement mechanism of this system. Our research helps to understand the enhancement mechanism of SERS based on 2D layered semiconductors. It not only provides a new way to effectively improve the SERS activity of a semiconductor SERS substrates but also helps to design more efficient and stable semiconductor SERS substrates for practical application.

## 2. Experiment

### 2.1. Synthesis of the SnSe_x_S_y_ Nanosheets

**Figure 1:**
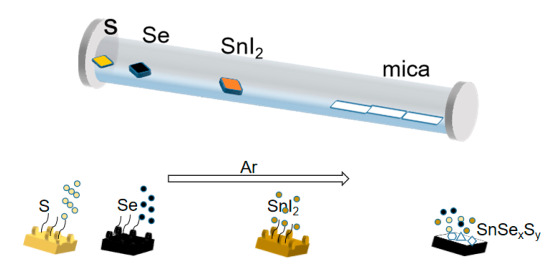
Experimental process for preparing SnSe*_x_*S*_y_* nanosheets.

Experimental process for preparing SnSe*_x_*S*_y_* nanosheets in Figure 1. The 2D SnSe*_x_*S*_y_* nanosheets were synthesized in a horizontal tube furnace (Lindberg/Blue M, Thermo Scientific, Asheville, NC, USA) with a quartz tube (in a diameter of 1 inch, and length of 20 inches) by CVD method. High purity SnI_2_ (99.999%, Alfa, Chengdu Alpha Metal Materials Co., Ltd., Chengdu, China) powder (0.05 g) was placed in the center of the furnace. Se (99.999%, Alfa, Chengdu Alpha Metal Materials Co., Ltd.) (0.4 g) with different mass of S (99.999%, Alfa, Chengdu Alpha Metal Materials Co., Ltd.) powder was placed upstream of the SnI_2_ powder at a distance of 13.5–14 cm to obtain different S-doped SnSe_2_ nanosheets. The corresponding sample numbers are presented in Table 1. Freshly cleaved mica substrates were placed downstream at a distance of 13–15 cm from the center. The growth temperature was kept at 600 °C for 10 min. Argon (Ar) as a carrier gas (during the growth of the material, we controlled the pressure to be in the near-atmospheric pressure range and used Ar as the transport gas such that the source powder can grow more stably on the mica and make it evenly doped) with a flow rate of 30 sccm was introduced into the quartz tube, and the pressure was maintained at atmospheric pressure during the whole experiment. After the reaction, the samples were allowed to cool to room temperature.

### 2.2. Characterization of SnSe_x_S_y_ Nanosheets

The chemical composition and *x* values of the samples were determined using an energy dispersive spectrometer (EDS, Sigma 500, Zeiss, Oberkochen, Germany). The crystalline structure of SnSe*_x_*S*_y_* was investigated by X-ray diffraction (XRD, Cu Kα, λ = 0.15406 nm, SMART APEX, Bruker Daltonics, Germany), transmission electron microscopy (TEM, JEM-2100, Japan Electron Optics Laboratory Co. Ltd., Tokyo, Japan), and X-ray photoelectron spectroscopy (XPS, Escalab 250, Thermo Fisher scientific, Waltham, MA, USA). The morphology and thicknesses were characterized by atomic force microscopy (AFM). AFM, Raman, and SERS measurements were performed using the integrated system from Horiba Jobin Yvon HR Evolution. Raman and SERS measurements were carried out using a 532 nm laser with a beam spot of ~2 μm. The incident laser power on the sample surface was kept below 0.5 mW to avoid any heating effects. The Raman spectra were collected using a 50× objective and integrated for 2 s. For SERS experiment, the aqueous solution of R6G ranging from 10^−4^ to 10^−8^ M was used. The prepared SnSe*_x_*S*_y_* substrates were dropped by R6G for various concentrations in aqueous solution, and dried at room temperature. In situ Raman mapping was performed on one of the SnSeS samples. A rectangular area (15 μm × 15 μm) of SnSe*_x_*S*_y_* was identified and mapped with 225 grid points. The Raman spectrum at each grid point was acquired using a confocal Raman microscope. An image of the identified area was created by employing statistical routines on the acquired spectral data.

## 3. Result and Discussion

The chemical compositions of the samples with different ratio of S and Se are presented in Table 1. It can be seen that as the ratio of the S in the source was increased, the *x* value of prepared samples continued to decrease from 1.14 to 0.64, which corresponds to the increase in S dopants in the samples. Meanwhile, the value of *x* + *y* decreased from 2.06 to 1.57 which means the ratio of Sn and S + Se of the samples changed from 1:1 to 2:3.

**Figure 2:**
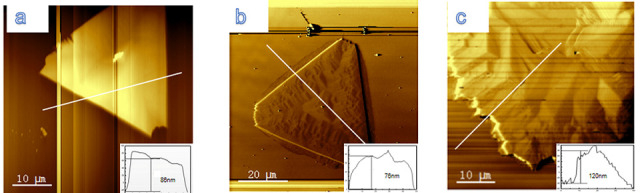
AFM images of (**a**) SnSe_1.14_S_0.92_, (**b**) SnSe_0.93_S_0.94_, and (**c**) SnSe_0.64_S_0.93_. The illustrations are the corresponding height profiles.

The amount of doping affects the morphology of the grown nanosheets. It can be seen from Figure 2a–c that the thicknesses of SnSe*_x_*S*_y_* nanosheets with different conditions range from 80 to 120 nm. With the increase in S dopant, the sample showed irregular truncated triangles (*x* = 1.14), regular truncated triangles (*x* = 0.93), and irregular polygons (*x* = 0.64). The growth of the nanosheets changed from 2D to 3D growth modes, and the sample showed a rougher and more undulating surface.

**Figure 3:**
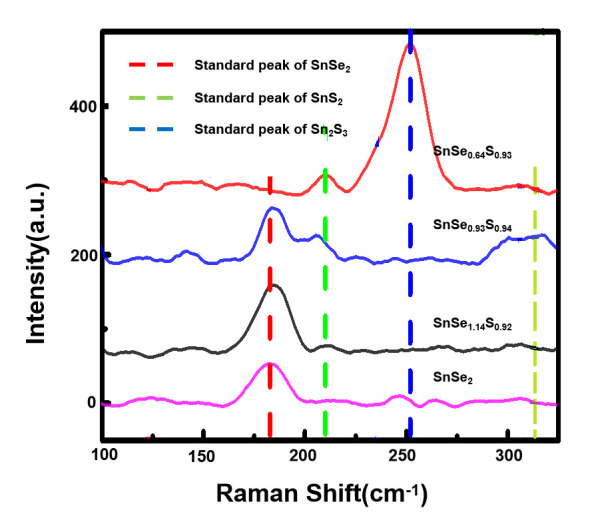
Comparison of Raman spectra of SnSe*_x_*S*_y_* nanosheets with increasing amounts of S dopant.

Raman spectra of the SnSe*_x_*S*_y_* nanosheets with various stoichiometric ratios are presented in Figure 3. A strong Raman peak at 183 cm^−1^ attributes to A_1g_ vibration modes of pure SnSe_2_ [22]. With the decrease in *x* value, a weak band appeared at 210 cm^−1^ which was attributed to the E_g_ vibration mode of SnS_2_. For Sample 3, the intensity of A_1g(Sn-Se)_ peak decreased, while the Raman peaks of SnS_2_ [23] at 215 and 313 cm^−1^ were significantly enhanced, and the Raman peak of Sn_2_S_3_ [24] appeared at 307 cm^−1^. This indicated that more S dopants replaced Se sites in the sample. As the *x* value dropped to 0.64, the Raman peak of A_1g(Sn-Se)_ and E_g(Sn-S)_ were relatively weak, while a strong Raman peak appeared at 251 cm^−1^, which was associated with Sn_2_S_3_.

**Figure 4:**
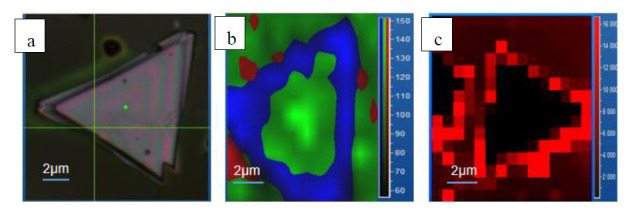
(**a**) SnSe_0.93_S_0.94_ light microscopy image. (**b**) Raman spectral intensity mapping for the A_1g_ mode (green) of SnSe_2_, and the A_1g_ mode (blue) of SnS_2_ for SnSe_0.93_S_0.94_ nanosheets. (**c**) The 2D SERS mapping of S-doped SnSe_2_ nanosheet.

To investigate the growth of S doping, the Raman frequency and intensity mapping of the A_1g_ vibration mode of SnSe_2_ (green) and SnS_2_ (blue) are shown in Figure 4b. The mapping of different A_1g_ vibration modes revealed the spatial distribution of the S dopants for SnSe_0.93_S_0.94_ nanosheets (We conducted XPS, TEM, XRD analysis on the surface structure and element composition of the material. The analysis results are shown in Figures S1–S3 in Supplementary Materials). It was found that the SnSe_2_-related A_1g_ mode mapping image was only distributed in the central of the nanosheets, and the most SnS_2_-related A_1g_ mode mapping image exhibited at the edge of the nanosheets, indicating a doping effect.

It is known that formation energy is very important for forming a stable alloy material. For pure SnSe_2_ and SnSe_0.93_S_0.94_ alloy, the formation energies (the formation energy is the minimum energy to produce a substance; the smaller the energy, the easier the corresponding substance is to synthesize) are about −0.5 and −4.1942 eV [25], respectively. In the CVD growth of S-doped SnSe_2_ nanosheets, Sn-Se bonds can be fabricated at first. As the electronegativity of S is higher than Se, S atoms can substitute Se in the SnSe_2_ nanosheet at a high temperature. Because of the 2D growth mode for SnSe_2_ on mica substrate, the unsaturated bonds at the edge of a nanosheet cause the substitution of S for Se (elements with a small atomic radius are easier to attract electrons and become electronegative ions; compared with the same main group, S has a smaller radius than Se, so it is more stable and easier to combine with Sn to form compounds). As the amount of S in the alloy increased, Sn-Se bonds become unstable and decompose, and Sn^2+^ and Sn^4+^ ions coexist in the alloy. The layered SnSe*_x_*S*_y_* alloys will be substituted by another phase material, Sn_2_S_3_, with orthorhombic structure. This results in an undulating surface of the alloy nanosheet. At the same time, it also caused the overall decrease of the value of *x* + *y* in the SnSe*_x_*S*_y_* compound. Hence, the 2D SnSe*_x_*S*_y_* alloys only can be formed within a suitable range of S-doped.

**Figure 5:**
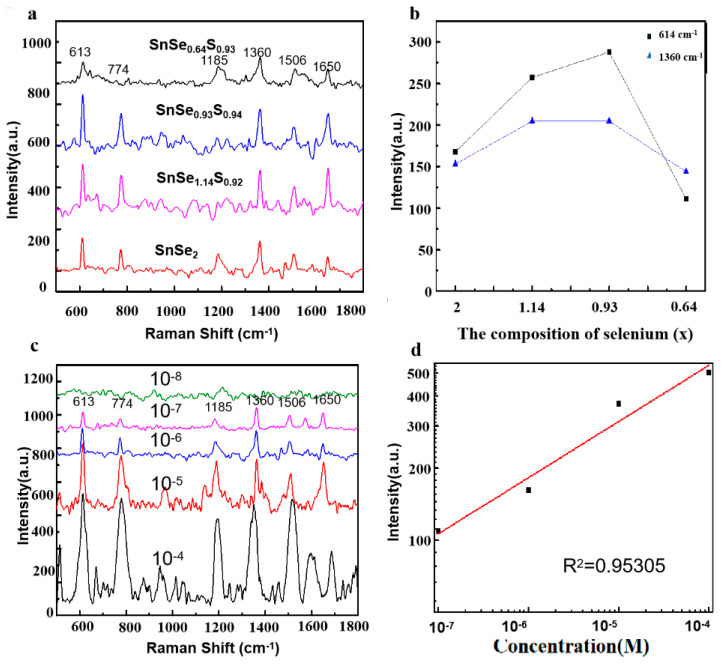
SERS spectra (**a**) and Raman intensities (**b**) of the peaks at 614 and 1360 cm^−1^ of 10^−6^ M R6G on different SnSe*_x_*S*_y_* nanosheets. SERS spectra (**c**) and the peak intensities (**d**) of the peaks at 614 cm^−1^ of R6G with concentration ranging from 10^−8^ to 10^−4^ M on SnSe_0.93_S_0.94_ nanosheets.

Figure 5a shows the comparison of Raman spectra of 10^−6^ M R6G on different S-doped SnSe_2_ nanosheets. The peaks at 614, 774, 1185, 1360, 1506, and 1650 cm^−1^ are the characteristic Raman peaks of R6G molecule. It can be seen that the intensity of the characteristic peaks of R6G increased first and then decreased with the increase in *x* value. For the sample with *x* = 0.93, by comparing the signals of the main peaks for 10^−6^ M R6G on the SnSe_0.93_S_0.94_ nanosheets with that on the pure SnSe_2_ nanosheets, it is observed that the magnitude of the Raman signal with different peaks is different, varying from 1.3 to 1.7 times. This is consistent with the chemical enhancement mechanism. For the sample with the best SERS activity (SnSe_0.93_S_0.94_), the limit of detection can reach 10^−7^ M for R6G aqueous solution. The relationship between the intensity of 614 cm^−1^ peak and the R6G concentration presented a consummate linear response with a high determination coefficient (R^2^ = 0.95305) in log scale. These results demonstrate that S doping has a significant effect on the SERS activity of the SnSe_2_ nanosheets for R6G molecule, and expands the application of SERS sensors based on SnSe*_x_*S*_y_* nanosheets in microanalysis and microdetection.

According to the Herzberg–Teller theory, PICT [26] processes will occur between the substrate and the adsorbed molecules when the energy levels of the highest occupied molecular orbital (HOMO) and lowest unoccupied molecular orbital (LUMO) of the adsorbed molecules match the conduction band (CB) and the valence band (VB) of the semiconductor substrate, respectively. The photon energy with 532 nm laser is suitable for the bandgap of R6G molecules (E_LUMO_ − E_HOMO_ = 2.3 eV). Therefore, once the energy of the incident photon (h*v*) is sufficient to excite the electrons in R6G from LUMO to HOMO, and CB to VB (h*v* = E_LUMO_ − E_HOMO_, or E_CB_ − E_VB_), resonance Raman scattering (RRS) will occur in the R6G molecule. For pure SnSe_2_, the E_CB_ of substrate is lower than the E_LUMO_ of molecule, while the E_VB_ of substrate is higher than the E_HOMO_ of molecule. Only a very few electrons of substrate were transferred to molecules through the excited coupling of the excited state of molecular and the CB of substrate. E_CB_ and E_VB_ of pure SnS_2_ are −4.14 eV and −6.40 eV [27]. Introduction of S dopant causes additional defect-related energy levels to reduce the VB level of SnSe_2_. When the VB of the substrate is close to the HOMO of R6G, the excited state electrons of the substrate will be excited to a higher energy level in CB and transferred to the LUMO of R6G through the coupling energy level between substrate and probe molecules. The charge transfer (CT) process will result in strong SERS activities for the probe molecules^22^. The SERS mapping was used to further illustrate the effect of doped that contribute to the SERS signal. Figure 4c represents 2D SERS mapping (at 614 cm^−1^) of R6G on S-doped SnSe_2_ nanosheet which was corresponding to the S-doped SnSe_2_ nanosheets shown in Figure 4. It was found that the SERS intensities of the 10^−6^ M R6G molecule have significant differences at different positions. The SERS signals of R6G are strong at the position where S doped, but weak at the center of the nanosheets (In order to prove the universal applicability of the samples, we detected biomolecules and pesticide residues, performed SERS detection of thiram and adenosine, and showed the Raman spectra with Figures S4 and S5). These results demonstrate that the SnSe_2_ nanosheets could be an excellent SERS-active substrate when doped by S.

## 4. Conclusions

The SERS characteristics of SnSe_2_ nanosheets can be improved by doped S to optimize the energy level matching between SnSe_2_ nanosheet substrate and the adsorbed molecules. This can be enhanced by the PICT between the substrate, and effectively improve the SERS activities of 2D semiconductor SnSe_2_ substrate. In our experiment, doped S caused the vibration coupling of the substrate and the adsorbed molecules, and affects the SERS activities of the SnSe_2_ nanosheets. The enhancement of Raman signals for 10^−6^ M R6G on few layers SnSe_0.93_S_0.94_ nanosheets can reach 1.3 to 1.7 times for different peak, and the LOD of R6G on SnSe_0.93_S_0.94_ substrate can be as low as 10^−7^ M. These results provide new ideas for us to study the mechanism of SERS process based on CM, and also provide a very important method for designing and improving the SERS characteristics of 2D semiconductor nanomaterial substrates.

## Figures and Tables

**Table 1:** Sample number and EDS results of SnSe*_x_*S*_y_* nanosheets samples.

No.	Ratio of S:Se in Source	Stoichiometry of Sample (%)			*x*	*y*	*x* + *y*
		Sn	Se	S			
0	—	33	67	—	2	—	2
1	1:20	32.78	37.27	29.95	1.14	0.92	2.06
2	1:10	34.80	32.24	32.95	0.93	0.94	1.87
3	7:10	38.91	25.07	36.02	0.64	0.93	1.57

## Supplementary Materials

The following are available online at https://www.mdpi.com/2079-4991/10/10/1910/s1, Figure S1: High-resolution XPS spectra of (a) pure R6G and R6G molecules on SnSe_0.93_S_0.94_ substrate, (b) Se 3d, (c) Sn 3d, and (d) S 3d for SnSe_0.93_S_0.94_ nanosheets and R6G/SnSe_0.93_S_0.94_ system. Figure S2: (a) The XRD result of the SnSe_0.93_S_0.94_ nanosheets grown on the mica substrate. (b) TEM and (c) HRTEM images of SnSe_0.93_S_0.94_ nanosheet. Figure S3: (a) and (c) are SEM and HRTEM images of SnSe_2_ nanosheet, (b) and (d) are SEM and HRTEM images of SnS_2_ nanosheet. Figure S4: SERS spectra of thiram. Figure S5: SERS spectra of adenosine.
