# Supplementary material for: Influence of SERS Activity of SnSe2 Nanosheets Doped with Sulfur
Source: Nanomaterials (Basel). 2020 Sep 24;10(10):1910. doi: 10.3390/nano10101910 (PMC7600932; doi:10.3390/nano10101910)
Supplement: Supplementary file 1 [file nanomaterials-10-01910-s001.pdf]

nanomaterials

Influence of SERS Activity of $SnSe_2$ Nanosheets Doped with Sulfur

Yuan Tian [1], Haonan Wei [1], Yujie Xu [1], Qianqian Sun [1] and Baoyuan Man [1,2] and Mei Liu [1,2*]

[1] School of Physics and Electronics, Shandong Normal University, Jinan 250038, China; 2018020524@stu.sdnu.edu.cn (Y.T.); 2018020530@stu.sdnu.edu.cn (H.W.); 2019020497@stu.sdnu.edu.cn (Y.X.); qianqiansun@sdnu.edu.cn (Q.S.); byman@sdnu.edu.cn (B.M.)

[2] Collaborative Innovation Center of Light Manipulations and Applications, Shandong Normal University, Jinan 250358, China

* Correspondence: liumei@sdnu.edu.cn

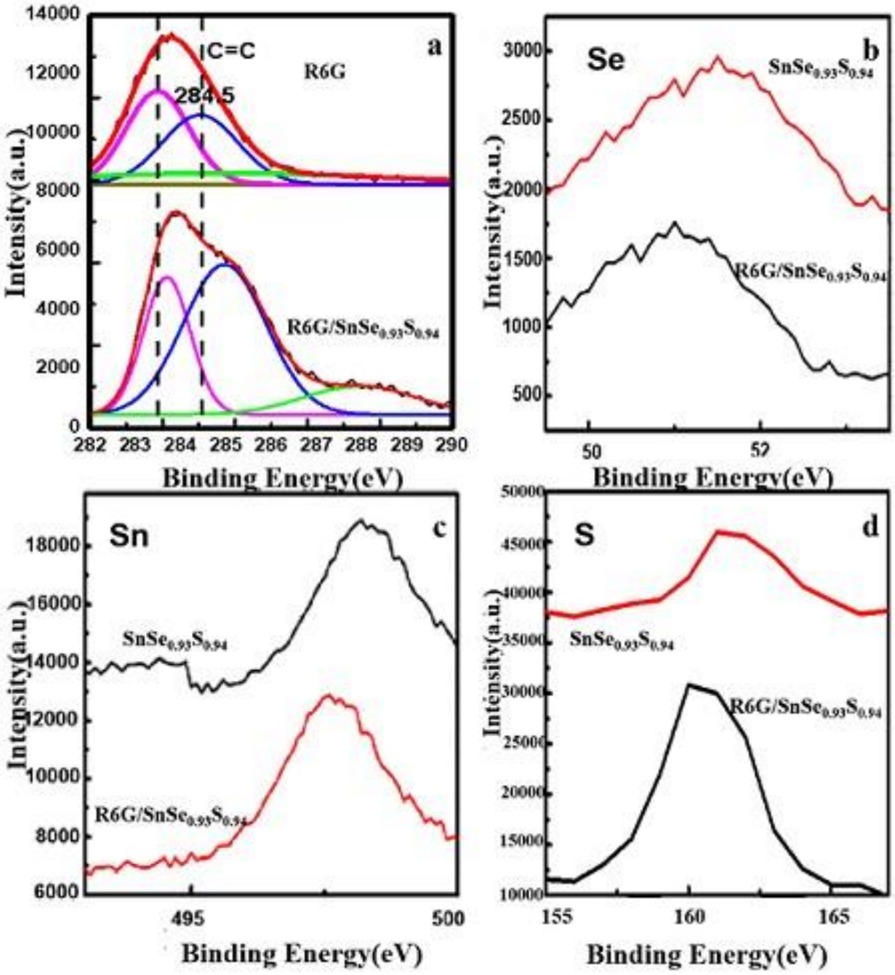

Figure 1. High-resolution XPS spectra of **(a)** pure R6G and R6G molecules on $SnSe_{0.93}S_{0.94}$ substrate, **(b)** Se 3d, **(c)** Sn 3d and **(d)** S 3d for $SnSe_{0.93}S_{0.94}$ nanosheets and R6G/$SnSe_{0.93}S_{0.94}$ system.

XPS analysis were carried out to confirm the chemical state of the R6G/$SnSe_{0.93}S_{0.94}$ system before and after SERS measurement. The bonding states of R6G was determined by peak fitting of C-C bond at 283.5 eV and C=C bond at 284.5 eV. The peaks at 51.7 eV (Figure S1b), 162 eV (Figure S1c), and 498 eV (Figure S1d) are Se 3d, S 3d, and Sn 3d in $SnSe_{0.93}S_{0.94}$ alloy which are assigned to Sn-Se and Sn-S bonds. For the R6G/$SnSe_{0.93}S_{0.94}$ system, all peaks shown in the figures have different shifts compared with the separated materials. The peak of R6G for SERS system was shifted to higher bond energies while the binding energies of $SnSe_{0.93}S_{0.94}$ alloy nanosheets for SERS system shifted to lower values, whic. indicates the variation in the electron affinity was caused by a charge transfer between the substrate and the probe molecules.

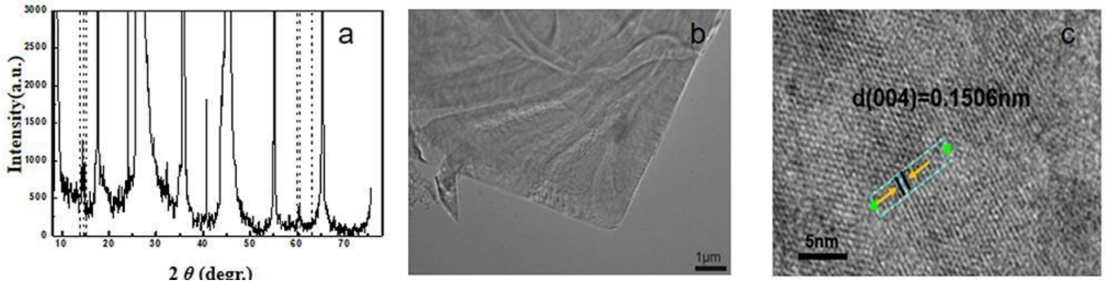

Figure 2. **(a)** The XRD result of the $SnSe_{0.93}S_{0.94}$ nanosheets grown on the mica substrate. **(b)** TEM and **(c)** HRTEM images of $SnSe_{0.93}S_{0.94}$ nanosheet.

XRD pattern of the SnSe0.93S0.94 nanosheets is shown in Figure S2(a). The prominent XRD peaks at 14.48° and 60.47° correspond to the (001) and (004) of the synthesized

SnSe0.93S0.94 nanosheets. They were different from those of the standard JCPDS file for SnSe2, appearing at 2θ = 14.414° (001) and 60.024° (004) (PDF#23–0602), and those for SnS2 appearing at 2θ = 15.029° (001) and 62.96° (004) (PDF#23–0677). The differences in the position of the diffraction peaks of the sample indicate that many Se positions in the subunits are occupied by S atoms. By the calculation formula of lattice spacing, 2dsin[illegible]=n[illegible], the lattice spacing of SnSe0.93S0.94 is 0.1529 nm.

Figure S2 (b) and (c) show a typical TEM and the corresponding HRTEM image of few layers SnSe0.93S0.94 nanosheets. The structure of the sample is relatively complete, and the specific surface area is large. The lattice plane spacing obtained from Figure 6(c) is about 0.1506 nm, which is still between SnSe2 (0.15400 nm) and SnS2 (0.14749 nm). The different values of the two sets may be derived from the error in the instrument and during the peak splitting process, which generally demonstrates the growth of nanosheets along the [001] direction.

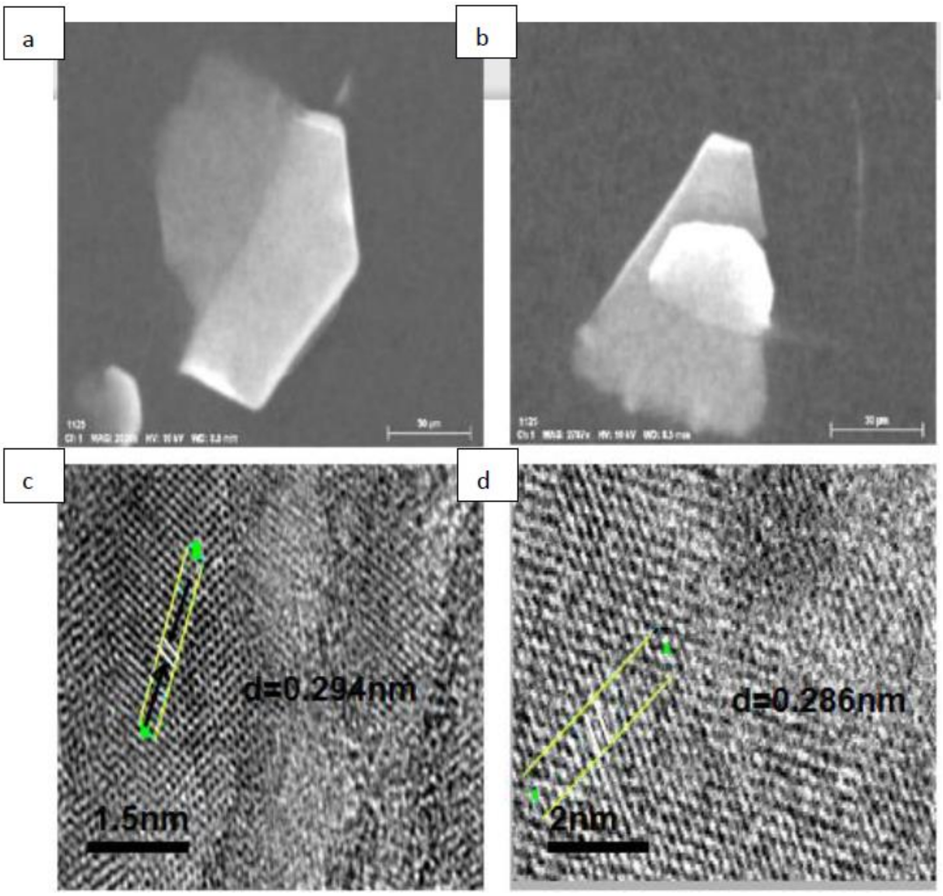

Figure S3. **(a)** and **(c)** are SEM and HRTEM images of $SnSe_2$ nanosheet, **(b)** and **(d)** are SEM and HRTEM images of SnS_2 nanosheet.

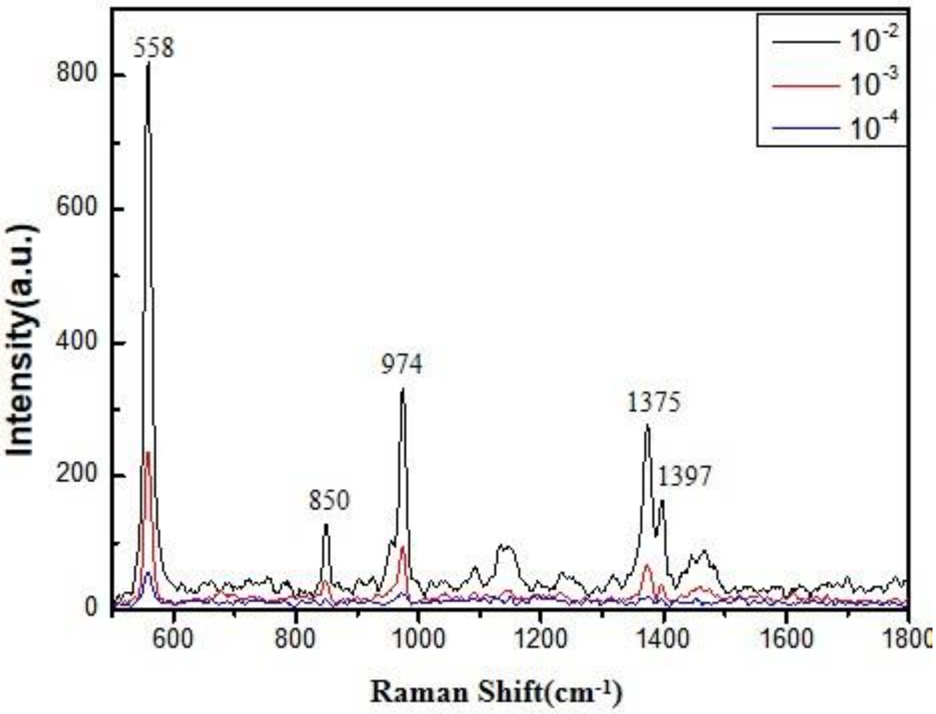

Figure S4. SERS spectra of thiram.

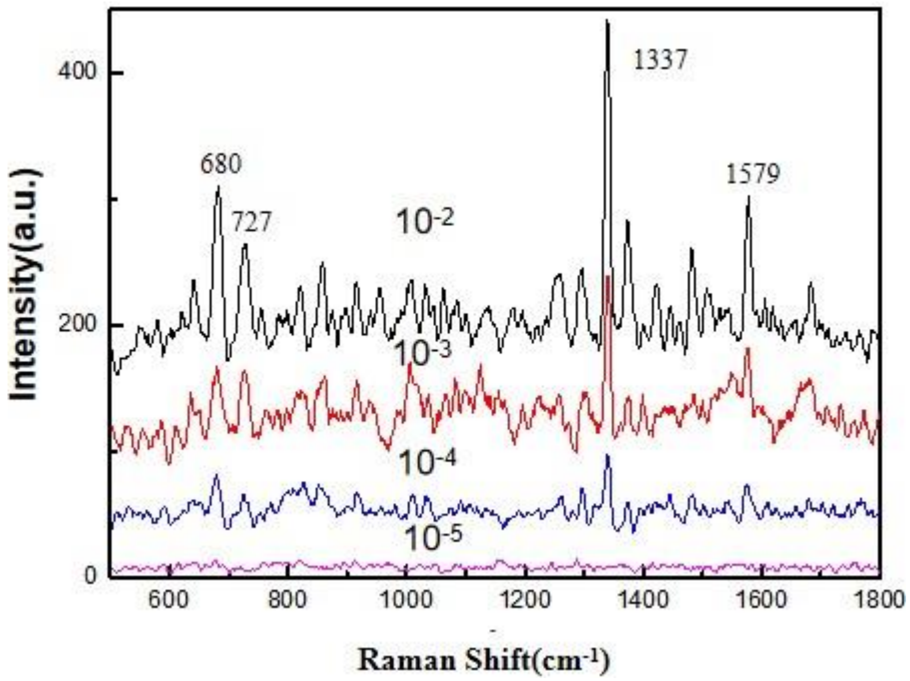

Figure S5. SERS spectra of adenosine.
